# Professor Dudley’s Operation for Stone by Lithotomy

**Published:** 1847-04

**Authors:** 


*Professor Dudley's Operation for Stone by Lithotomy.*—(Western Lancet, Jan., 1847.) Professor Dudley has recently performed the operation for stone in several instances, and with his usual success. He has now performed the operation on *one hundred and eighty-nine persons*, of whom one hundred and eighty-four have recovered. The last case from which we received particular information, recovered in less than a week. Twelve hours after the operation, the urine passed by the wound for the last time, and union by the first intention took place perfectly.

This remarkable success has not been attained by a *selection* of cases; on the contrary, we have the best authority for the declaration that he has operated on nearly every case that has been presented. He performs the lateral operation, and always employs the gorget.—*Southern Journal of Medicine and Pharmacy.*

